# Correlation of serum cartilage oligomeric matrix protein with knee osteoarthritis diagnosis: a meta-analysis

**DOI:** 10.1186/s13018-018-0959-y

**Published:** 2018-10-19

**Authors:** Xiaoyang Bi

**Affiliations:** Department of Orthopedic Medicine, Tianjin Hospital, No 406 JieFangNan Road , Hexin District, Tianjin City, 300211 China

**Keywords:** Knee osteoarthritis, Cartilage oligomeric matrix protein, Kellgren-Lawrence

## Abstract

**Background:**

The measurement of cartilage oligomeric matrix protein (COMP) has become a novel way for the diagnosis of knee osteoarthritis (OA). However, no conclusive correlation has been drawn between COMP and knee OA. The purpose of this study was to examine the utility of serum COMP as biomarker for knee OA and its relation with disease severity.

**Methods:**

A systematic search on PubMed, ScienceDirect, and EMBASE was conducted in January 2018 using certain keywords. Initial search yielded a total of 285 publications, and 35 articles were reviewed in full-text. Eventually, nine studies were included in the analysis. All the retrieved studies used Kellgren-Lawrence (K-L) classification for knee OA and provided available data of serum COMP in OA patients and healthy controls. Sensitivity analysis was performed by removing one study result at a time to detect the impact of each study have on the overall effect and to test the stability of the cumulative result. Subgroup study based on K-L grade system was also conducted to disclose the correlation between serum COMP and knee OA disease severity.

**Results:**

Pooled analysis of nine studies demonstrated a significant elevation of serum COMP in knee OA patients (SMD 0.81, [95% CI, 0.36, 1.25], *P* = 0.0004) compared with controls. In comparisons between K-L 1–4 and controls, significantly higher serum COMP was detected in all three subgroups except K-L grade 1 versus control. Comparisons among K-L grades 1–4 revealed significantly higher serum COMP levels in patients with more serious than less serious disease stage. However, the elevation in patients with K-L grade 3 did not reach statistical significance when compared with K-L grade 1 patients.

**Conclusion:**

The overall analysis showed significantly higher serum COMP in knee OA patients compared to controls which indicate the potential ability of serum COMP in differentiating knee OA patients from healthy subjects. Pooled statistic of our meta-analysis showed that serum COMP levels were effective in distinguishing patients with K-L ≥ 2.

## Background

Osteoarthritis (OA) is one of the most common joint diseases worldwide, affecting approximately 9.6% of men and 18% of women in the elderly [1]. It is characterized by progressive destruction of the articular cartilage and substantial abnormalities in the subchondral bone, ligaments, synovial membrane, articular capsule, and periarticular muscles. OA can be triggered by various factors like inflammation, physical injury, and other metabolic causes [2]. A number of environmental risk factors such as obesity and trauma can also initiate diverse pathological pathways which may eventually lead to OA [3].

Until now, the most reliable method for OA assessment is joint space width (JSW) measurement using radiography [4, 5]. However, since the disease is initiated long before the plain X-rays can be detected, irreversible joint damages have often already occurred at the time radiological diagnosis is established. Therefore, more sensitive techniques for early diagnosis of OA are needed. Magnetic resonance imaging (MRI) is well-established for this purpose. However, there are still obstacles in availability and cost of this imaging approach [6]. Biomarkers, molecules that are secreted into biological fluids during matrix metabolism of articular cartilage, subchondral bone, and synovial tissue, have received increased research attention for the diagnosis of OA. A variety of biomarkers, such as proinflammatory and anti-inflammatory cytokines, catabolic enzymes, and markers of cartilage and bone turnover, can be applied to OA diagnosis. According to the “BIPED” classification, each biomarkers can be classified to one or more of the following five categories: burden of disease, investigative, prognostic, efficacy of intervention, and diagnostic [7]. A study reviewing the status of available biochemical markers for OA suggests that cartilage markers are the most extensively investigated and well-performed type in comparison with the bone or synovial tissue biomarkers [8].

Cartilage oligomeric matrix protein (COMP), a 524-kd pentameric glycoprotein related to the thrombospondin family, is one of the cartilage markers [9]. COMP is found predominantly in cartilage, and recent studies have demonstrated that COMP expression can also be identified in other structures such as the ligaments, tendons, menisci, and synovial membrane [10, 11]. Since its appearance, the diagnostic value of serum COMP for OA as well as its correlation with disease progression and severity has been frequently and broadly assessed [9, 12–14]. However, the efficiency of serum COMP as biomarker for OA diagnosis is still in controversy. Several previous studies suggested that serum COMP had the ability to distinguish OA patients from healthy controls, as significant higher serum COMP levels were detected when compared with controls [15, 16]. Some research suggested that more rapid knee, hip, or hand joint destruction would occur in patients with higher levels of COMP in serum in comparison to that in patients with lower levels [17, 18]. Other studies, however, found no correlation between serum COMP and OA presence at all [19, 20]. One possible reason for such discrepancy could be the limited statistical power of studies due to their relatively small sample sizes.

Thus, by collecting and combining all available data, the primary objective of the present meta-analysis is to assess the diagnostic performance of serum COMP as biomarker for knee OA as well as the correlation between serum COMP levels and knee OA disease severity classified by Kellgren-Lawrence (K-L) grade.

## Methods

### Search strategy

Electronic databases, PubMed, ScienceDirect, and EMBASE, were systematically searched for relevant publications till January 2018. The search terms were as follows: osteoarthritis, cartilage oligomeric matrix protein, serum, knee OA, diagnosis, and all of the combinations. Reviewer screened all abstracts retrieved from the initial search results. Study was reviewed in full-text if it is relevant to our topic or the abstracts did not provide enough information to include or exclude the study from the review. Further manual search of all reference lists and other relevant meta-analysis were conducted for additional studies which were not included in the original search. There was no restriction on studies in terms of their year, region, or language of publication. However, all the selected non-English articles must contain an English abstract.

### Eligibility criteria

All the studies need to fulfill the following criteria to be included in the meta-analysis:

- Studies involved patients with radiographic diagnosis of knee OA.
- Studies with a disease free control group for comparison.
- The severity of knee OA was graded using K-L classification.
- Studies provided extractable serum COMP levels in both knee OA and control group.
- All enrolled participant were adult (age > 18).
- Levels of serum COMP were quantified using enzyme-linked immunosorbent assay (ELISA) regardless of type and manufacture company.

Studies that did not match the above requirements, review papers, case reports, and other non-related studies were excluded. We only included publications that applied K-L severity grade system for knee OA. Studies that used other OA severity classification systems were excluded to guarantee consistent comparisons across studies.

### Data extraction

Reviewer inspected and extracted all the relevant data according to predefined form. In case of discrepancies, a second reviewer was reached and solved them. The outcome of interest for this study was differences of serum COMP levels between the knee OA patients and controls. A majority of studies reported serum COMP in unit of measure nanograms per milliliter or milligram per milliliter [9, 18, 19, 21–23], but some studies used the unit units per liter [17, 20, 24]. For this analysis, serum COMP in all units of measure was extracted, and data were combined using a standardized mean differences (SMD) model. Other study characteristics, including sample size, number of woman patients, patients’ body mass index, number of patients in different K-L grade group, type and manufacture information of the ELIAS kits, and the region in which the study take place, were also extracted.

### Statistical analysis

Inter-group analysis of serum COMP was based on the difference in the levels of COMP between knee OA patients and healthy controls. Statistical analysis was performed using chi-squared test and manifested by forest plot where *Q* and *I*^2^ were presented. A random effects model was used when considerable heterogeneity (*I*^2^ > 50%) existed among studies. A fixed-effects model was applied when *I*^2^ < 50%. Since the included studies measured the levels of serum COMP with different scales, pooled results of this meta-analysis were calculated using SMD with 95% confidence intervals (CIs). Sensitivity analysis were performed by removing one study result at a time to determine the influence of individual study have on the overall estimate and to test the stability of the cumulative result across the included studies [25]. Subgroup analysis based on different unit of measure was performed to explore the source of heterogeneity across study, and outcomes were presented as mean difference (MD) with 95% CI. To further reveal the mechanism of serum COMP in different OA severity, subgroup analysis was also conducted by comparing serum COMP levels among different K-L grades. All statistical analyses were processed using Review Manager (Version 5.3. Copenhagen: The Nordic Cochrane Centre, The Cochrane Collaboration, 2014). We considered *P* value < 0.05 as statistically significant.

## Results

### Study selection

**Fig. 1:**
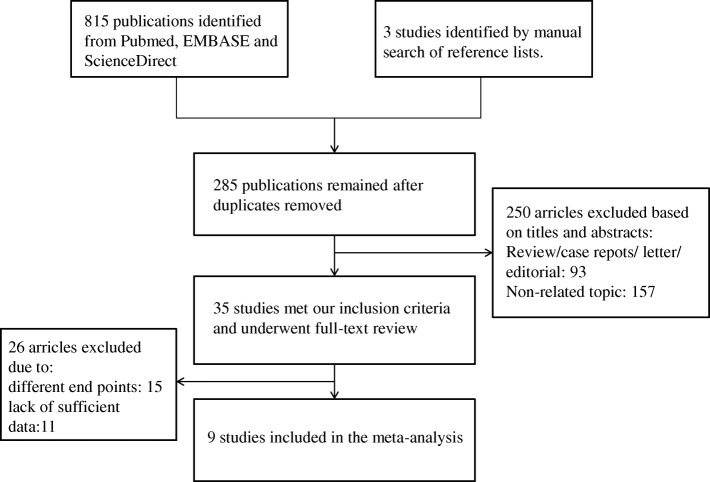
Flow diagram of study selection

The electronic databases and manual cross-checking of reference lists identified 285 articles for the initial review. After examining all the tiles and abstracts by one reviewer, 35 studies satisfied the most crucial and basic criterion which was assessing the efficiency of serum COMP as a biomarker for knee OA remained and went through full-text review. Excluding studies that did not use K-L grade for OA classification and studies that did not provide necessary data, nine studies were included in this meta-analysis. Figure 1 presented the flow diagram of study selection.

### Study description

Our analysis only included studies that involved patients with radiological defined knee OA classified by K-L severity grade and measured COMP concentrations in serum. The variables in terms of joint and type of OA, source of COMP, and OA classification system were restricted. Other characteristics, such as sample size, patients’ age, body mass index (BMI), and ethnic groups, were varied from individual study. Eventually, 9 studies with a total of 1694 participant were included in this study [9, 17–24]. Overall, the sample size of the enrolled studies ranged from 48 to 769 patients. Selected participants were more than 20 years old. Most of the study applied a sandwich ELISA kits while competitive and two-site ELISA were also used by one study respectively [9, 24]. Manufacture of the ELISA assays were also varied from study to study, and several trials applied an ELIAS kit modified by authors [9, 18, 22]. For outcome measurement, three studies expressed serum COMP in the unit units per liter [17, 20, 24]. Other six used the unit of measure nanograms per milliliter or milligram per milliliter. Table 1 summarized all relevant detailed characteristics of the included studies.

**Table 1:** Characteristics of included studies for COMP

Author	Group	*N*	Age (years)	Female (*n*)	BMI (kg/m2)	Region	K-L 0~4 of patients (*n*)	ELISA manufacture information	Type of ELISA
Clark1999	Control	148	60 ± 20	144		USA	K-L 0 = 148, K-L2 = 109 K-L 3,4 = 34	In-house method	Competitive
	OA	143	60 ± 20						
Das Gupta E2017	Control	30	62.5 ± 12	15	25.01 ± 6.31	Malaysia	K-L 2 = 30 K-L 3 = 23 K-L 4 = 7	R&D Systems	Solid-phase sandwich
	OA	60	57.5 ± 9	46	26.72 ± 5.35				
Fernandes2007	Control	40	53.8 ± 8.5	24		Brazil	K-L 0,1 = 40 K-L 2–4 = 75	AnaMar Medical (Uppsala, Sweden)	Sandwich
	OA	75	56.6 ± 7.6	51					
Jordan JM2003	Control	302	63.3 ± 10.8	258	31.5 ± 7.4	USA	K-L 0 = 302, K-L 2 = 313, K-L 3 = 110, K-L 4 = 44	AnaMar Medical (Lund, Sweden)	Sandwich
	OA	467	60.6 ± 9.6	190	28.9 ± 6.1				
Li2012	Control	35	53 ± 12.53	19	21.8 ± 4.9	China	K-L 1 = 28, K-L 2 = 36, K-L 3 = 27, K-L 4 = 24	AnaMar Medical AB (Lund, Sweden)	Sandwich
	OA	115	55 ± 13.32	60	22.3 ± 5.7				
Mündermann A2009	Control	41	57.5 ± 7	20	25.7 ± 4.2	USA	K-L 1 = 11, K-L 2 = 7, K-L 3 = 12, K-L 4 = 12	AnaMar Medical AB (Lund, Sweden)	Sandwich
	OA	42	60.7 ± 8.6	22	27.0 ± 3.8				
Author	Group	N	Age (years)	Female (*n*)	BMI (kg/m2)	Region	K-L 0~4 of patients (n)	ELISA manufacture information	Type of ELISA
Senolt L2005	Control	38	58.3 ± 9.1	23		Czech Republic	K-L 1 = 2, K-L 2 = 18 K-L 3 = 14, K-L 4 = 4	In-house method	Sandwich
	OA	38	64.1 ± 10.1	25					
Sowers MF2009	Control	36	47.5 ± 2.6	36	29.7 ± 6.2	France	K-L 0,1 = 36 K-L 2 = 16 K-L 3,4 = 20	AnaMar Medical (COMPTM ELISA kit)	A two-site ELISA
	OA	36	47.5 ± 2.6	36	39.15 ± 8.22				
Wakitani2007	Control	24	20–54	8		Japan	K-L 1 = 7, K-L 2 = 4 K-L 3 = 6, K-L 4 = 7	Kamiya Biomedical Company, Seattle, WA, USA	Sandwich
	OA	24	40–80	20					

*COMP* cartilage oligomeric matrix protein, *K-L* Kellgren-Lawrence classification, *ELISA* enzyme-linked immunosorbent assay

### Correlation between serum COMP levels and knee OA

**Fig. 2:**
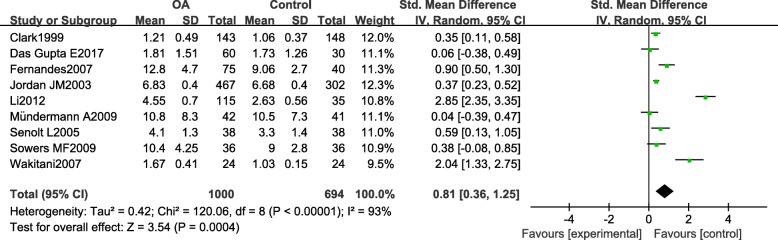
Forest plot of the standardized mean difference of serum COMP in patients with knee OA compared with controls. A positive standardized mean difference represents a higher serum COMP levels in the knee OA patients compared with controls

Pooled results of nine studies revealed a significant elevation of serum COMP in OA patients compared with healthy controls (SMD 0.81, [95% CI, 0.36, 1.25], *P* = 0.0004, Fig. 2). Considering high heterogeneity existed among studies (*I*^2^ = 93%), sensitivity analysis was also conducted. The pooled SMD ranged from 0.51 to 0.90 when removing each given study, and all the outcomes still remained statistical significant. The forest plot figure and results of the sensitivity analysis indicated that the study of Li might be the source of heterogeneity. When omitting the study result of Li et al., the pooled SMD changed from 0.81 (95% CI, 0.36, 1.25) to 0.51 (95% CI, 0.25, 0.78) with *I*^2^ value dropped from 93% to 79%. Subgroup analysis based on different measure of units was also performed. Results showed increased serum COMP in knee OA patients compared to controls in both units per liter unit (MD 2.13, [95% CI, 0.11, 4.16], *P* = 0.04, Table 2) and microgram per milliliter unit subgroups (MD 0.73, [95% CI, 0.02, 1.44], *P* = 0.04, Table 2).

**Table 2:** Subgroup analysis of serum COMP with different unit of measure and disease severity

Subgroups	Number of study	Mean difference	95% CI	*P*
Unit of measure				
U/L	3	2.13	0.11, 4.16	0.04
μg/ml	6	1.00	0.37, 1.63	0.002
Disease severity				
K-L 1 VS K-L 2	3	0.79	0.34, 1.25	0.0007
K-L 1 VS K-L 3	3	1.08	0.44, 2.60	0.16
K-L 1 VS K-L 4	3	1.61	0.57, 2.65	0.002
K-L 2 VS K-L 3	6	0.44	0.04, 0.85	0.03
K-L 2 VS K-L 4	7	0.61	0.17, 1.05	0.007
K-L 3 VS K-L 4	5	0.41	0.15, 0.68	0.002

*K-L* Kellgren-Lawrence classification, *CI* confidence intervals

### Correlation between serum COMP levels and OA severity

**Fig. 3:**
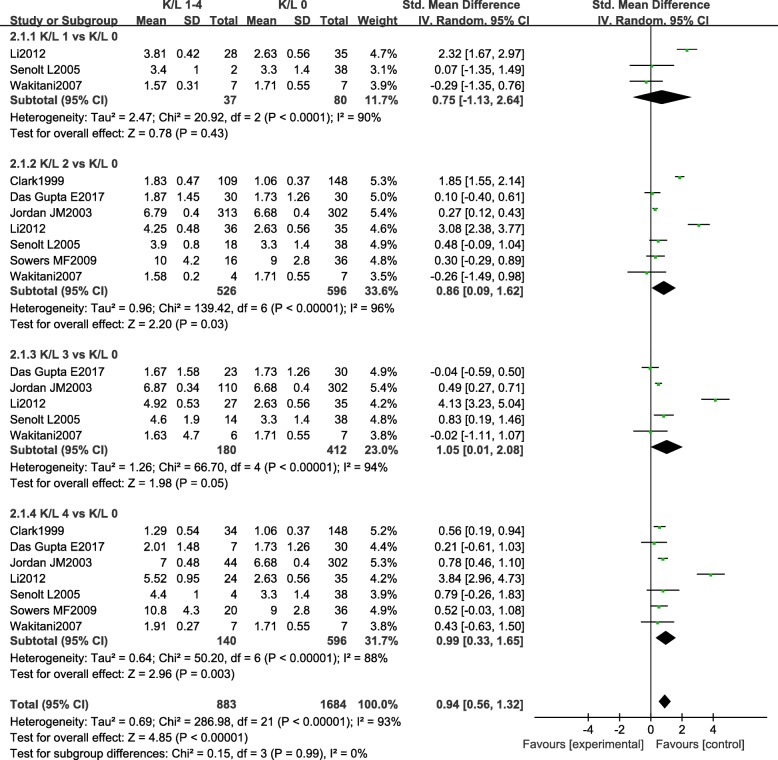
Forest plot of the standardized mean difference of serum COMP in patients with K-L grades 1–4 knee OA compared with controls. A positive standardized mean difference represents a higher serum COMP levels in the knee OA patients compared with controls

For studies providing serum COMP data in different K-L grades, comparisons were performed between K-L grades 1–4 and controls. Significant differences in serum COMP were observed comparing patients with K-L grades 2–4 with healthy controls (K-L 2: SMD = 0.86, 95% CI, 0.09, 1.62, *P* = 0.03; K-L 3: SMD = 1.05, 95% CI, 0.01, 2.08, *P* = 0.05; K-L 4: SMD = 0.99, 95% CI, 0.33, 1.65, *P* = 0.003) (Fig. 3) Sensitivity analysis further showed that the study of Li might be the reason of high heterogeneity. When removing the result of Li, the outcomes still showed significant higher serum COMP levels in knee OA patients with K-L grades 2–4 compared with controls, while the *I*^2^ value changed from > 90% to < 50%. Patients with OA of K-L grade 1 tended to have higher serum COMP concentrations than healthy participants, although the differences were not significant (Fig. 3).

Comparisons within different K-L grades were also conducted. In the comparison between K-L grades 2–4 and K-L grade 1, significant differences were found in K-L grade 2 and K-L grade 4 compared with K-L grade 1. Serum COMP levels tended to be higher in patient with K-L grade 3 than with K-L grade 1; however, the result did not reach statistical significance. A significant elevated serum COMP levels were found in subgroups K-L grades 3–4 versus K-L grade 2 and K-L grade 4 versus K-L grade 3. Pooled statistics revealed that serum COMP levels had a tendency to increase as OA symptom became more serious (Table 2).

## Discussion

Biomarkers have risen as a non-invasive and more sensitive measurement in detecting subtle changes in bone, cartilage, and synovial tissues, which is more capable of diagnosing early sight of OA [26–28]. The measurement of serum COMP becomes popular among researchers as a potential indicator for OA [8, 9, 29, 30]. It is believed that changes in serum COMP can reflect changes in cartilage breakdown [10, 31]. Some researchers also put forward that the presence of OA might induce more active cartilage turnover, resulting in a greater percentage increase in serum COMP levels [32]. However, the conclusions from previous investigations were differed from study to study, and a single study could not provide enough evidence to confirm to usefulness of serum COMP in diagnosing knee OA. Thus, we performed the current meta-analysis to systematically combine all available information and assesse the correlation of serum COMP levels with knee OA and disease severity. The combined result from 9 studies involved 1000 knee OA patients and 694 healthy participants demonstrated a significant elevation in serum COMP levels in knee OA patients compared to controls. Although considerable heterogeneity existed, sensitivity analysis still revealed significantly higher levels of serum COMP in knee OA group compared to controls when we removed each study result. Also, we found that the study of Li might be the source of heterogeneity as the *I*^2^ value dropped when we omitted its outcome. Comparisons were made between Li et al. and other included studies. However, the study of Li did not differ much from the others in study design, patients demographic, and biomarker quantify method, except that it was the only included study assessed serum COMP in Chinese patients and the article was published in Chinese. The different body structure of participant or the differential expression of the Chinese language might be the distributions of heterogeneity. Future analysis might need to restrict the population and language of the included study. Nevertheless, our finding supported the perspective that serum COMP levels could serve as effective biomarker for diagnosing knee OA.

As part of the inclusion criteria, the present analysis must include study that used K-L severity grade system for the classification of knee OA patients. This classification system was chosen because it was the most widely and commonly used classification tool in clinical practice and in research, especially in grading OA [33]. To further prove whether serum COMP was correlated with knee OA disease severity, subgroup analyses between K-L grades 1–4 and control group and comparisons among K-L grades 1–4 were carried out. Significant differences were found in all the subgroups except the comparison of K-L grade 1 versus control and K-L grade 3 versus grade 1. Although the result of the two subgroups did not reach statistical significance, data still showed that compared with patients with a lower severity, patients with higher disease severity had higher serum COMP. However, only three studies provided COMP levels in K-L grade 1 patients [21–23], the limited data might be insufficient to define the relationship between K-L grade 1 and other groups. These uncertain results might also cause by the different definitions for each of the K-L classifications used to assess OA severity in each study. A recent review of K-L classifications used in published study reported grade 2 definitions are different throughout the reported literature [34]. Only four studies recruited in this study provided specific K-L classifications. Future investigation should confirm the definition of K-L grade system allowing better comparisons to be made across the studies. According to the outcome of our analysis, we observed a potential correlation between serum COMP and knee OA severity. The levels of serum COMP trend to rise as the disease become more serious. However, whether serum COMP is useful in diagnosing early knee OA need further evidence.

Previous research suggested that ethnic differences could affect the symptom manifestations in knee OA population [35]. A study by GANDHI investigating the influence of ethnic differences on joint pain and function in knee OA patients reported that joint pain and dysfunction were greater in Asian patients than in Caucasians [36]. In assessing the correlation between serum COMP and knee OA, researchers also point out the influence of ethnicity have on the levels of COMP. In the study of Jordan et al., higher levels of serum COMP were found in African American women compared with that in Caucasian women in both controls and knee OA patients [18]. Three of the included studies assessed serum COMP in specific ethnic group, Caucasian, Brazilian, and Malaysian [17–19]. Results showed that serum COMP was significantly elevated in Caucasian and Brazilian patients versus controls [9, 17]. Whereas no difference in serum COMP levels between healthy controls and knee OA patients were found in Malaysian population [19]. Notably, in overall analysis, sensitivity analysis showed that Li et al. and Wakitani et al. affected mostly on the overall effect [21, 23]. When removing the data of Li and Wakitani, the SMD effect size dropped from 0.81 to 0.51, and from 0.81 to 0.67. Interestingly, both studies were performed in Asian population. This observation put us to further consideration that the diagnostic performance of serum COMP might be preferable in certain ethnic group. However, comparisons among different ethnicities could not be conducted in our analysis due to the limited and insufficient information. These factors should be considered in the derivation of standards using this, and possibly other, potential biomarkers of OA.

There are still some limitations. First of all, although sensitivity and subgroup analyses were performed to confirm the statistic power of the study, the effect of heterogeneity still cannot be eliminated completely. We assumed that the inclusion of small sample size studies might be the reason causing inconclusive and imprecise results. However, only nine publications met all our inclusion criteria, it is impossible to omit the outcomes of small sample size studies in this meta-analysis. Furthermore, the use of different ELISA kits might be another reason causing heterogeneity and discrepancy. Some researchers put forward that certain ELISAs were more appropriate for detecting human serum COMP than others. A study comparing three ELISA kits for measuring COMP in serum concluded that an in-house method utilizing MAb’s 16F12 and 17C10 and the Biovendor kit (Modrice, Czech Rep.) were more suitable for detecting serum COMP than Anamar kit (Gothenburg, Sweden) [37]. A previous meta-analysis reported that the ELISA kit manufactured by Kamiya Biomedical Company was preferable than other ELISA kits in the measurement of serum COMP [30]. However, due to the diverse ELISA kits involved in our study, subgroup analysis could not be performed to prove which ELISA kit is the best choice. Yet, by examining the results of the nine enrolled studies, we observed that two of them revealed no significant difference in serum COMP levels between knee OA patients and controls [19, 23]. These two studies applied a R&D Systems ELISA and a Kamiya Biomedical ELISA respectively, while other studies utilized in-house method or AnaMar Medical kit manufactured by different company. However, the direct comparison among studies is less rigorous. To ascertain the best ELISA kits for assessing serum COMP, the only way is to compare the serum sample quantified by the each technique from the same subjects. Future experiments should investigate in this field. Our study only estimates the diagnostic value of serum COMP in knee OA. The effectiveness of synovial fluid COMP in predicting knee OA and the usefulness of serum COMP in detecting other joints of OA remain to be established.

## Conclusion

The overall analysis revealed a significant elevation of serum COMP in knee OA patients compared to controls. The overall effect showed that serum COMP had the potential to differentiate knee OA patients from healthy subjects. In assessing the correlation between serum COMP and knee OA disease severity, although the result of K-L 1 versus control and K-L grade 3 versus K-L 1 did not reach statistical significance, serum COMP still showed elevation toward patients with more serious OA stage. Pooled statistic of our meta-analysis showed that serum COMP levels were better in distinguishing patients with K-L grades ≥ 2. Future rigorous prospective study is required in order to strengthen our findings.

## Abbreviations

CI: Confidence intervals
COMP: Cartilage oligomeric matrix protein
ELISA: Enzyme-linked immunosorbent assay
JSW: Joint space width
K-L: Kellgren-Lawrence
MD: Mean differences
OA: Osteoarthritis
SMD: Standardized mean differences
